# On the Proximate Cause and Specific Remedy of Tuberculosis

**Published:** 1858-09

**Authors:** 


### *On the Proximate Cause and Specific Remedy of Tuberculosis.* By Dr. John Francis Churchill.

The following is the abstract of a paper which was laid before the Academy of Medicine at Paris, on the 21st of July, 1857:

The total number of cases of phthisis treated by the author, amounts to 35. All were in either the second or the third stage of the complaint—that is, they had either softened tubercles or cavities in the lungs. Of these 9 recovered completely, the physical signs of the disease disappearing altogether in 8 out of that number; 11 improved considerably, and 14 died; 1 still remains under treatment.

The proximate cause, or at all events, an essential condition of the tubercular diathesis, is the decrease in the system of the phosphorus which it contains in an oxygenizable state.

The specific remedy of the disease consists in the use of a preparation of phosphorus, uniting the two conditions of being in such a state that it may be directly assimilated, and at the same time at the lowest possible degree of oxydation.

The hypophosphites of soda and lime are the combinations which hitherto seem best to fulfill these two requisites. They may be given in doses varying from ten grains to one drachm in the twenty-four hours. The highest dose which I have been in the habit of giving to adults is twenty grains.

The effects of these salts upon the tubercular diathesis is immediate, all the general symptoms of the disease disappearing with a rapidity which is really marvellous.

If the pathological deposit produced by the dyscrasy is of recent formation, if softening has only just set in and does not proceed too rapidly, the tubercles are absorbed and disappear; when the deposit has existed for a certain time, when the softening has attained a certain degree, it sometimes continues in spite of the treatment, and the issue of the disease then depends upon the anatomical condition of the local lesion, on its extent, and upon the existence or non-existence of complications. The author has made numerous attempts to modify the local condition of the lungs by the inhalation of different substances, but has never obtained any satisfactory result independent of what was to be attributed to the specific treatment. The hypophosphites of soda and lime are certain prophylactics against tubercular disease.

The physiological effects which he has observed to be produced by the use of the hypophosphites of soda, lime, potash and ammonia, show these preparations to have a twofold action. On the one hand they increase the principle, whatever that may be, which constitutes nervous force; and on the other, they are the most powerful of hæmatogens, being infinitely superior to all medicines of that class hitherto known. They seem to possess in the highest degree all the therapeutical properties formerly attributed by different observers to phosphorus itself, without any of the danger which attends the use of that substance, and which has caused it to be almost forgotten as a medical agent. The different preparations of hypophosphorous acid will, according to these views, occupy one of the most important places in the Materia Medica.—*Dublin Hospital Gazette*, from *Ranking's Abstract of Med. Sciences.*

